# Air particulate matter SRM 1648a primes macrophages to hyperinflammatory response after LPS stimulation

**DOI:** 10.1007/s00011-018-1165-4

**Published:** 2018-06-19

**Authors:** Anna Gawda, Grzegorz Majka, Bernadeta Nowak, Małgorzata Śróttek, Maria Walczewska, Janusz Marcinkiewicz

**Affiliations:** Chair of Immunology, Jagiellonian University Medical College, Kraków, Poland

**Keywords:** Air pollution, SRM 1648a, Particulate matter, Inflammation, Macrophage priming

## Abstract

**Objective:**

Exposure to air particulate matter (PM) is associated with chronic inflammatory and autoimmune diseases. Macrophages are responsible for the regulation of chronic inflammation. However, whether PM affects macrophage polarization remains unclear. The aim of this study was to evaluate whether nontoxic concentrations of urban PM are able to prime macrophages to altered inflammatory response upon LPS challenge.

**Methods:**

We used two forms of the urban particulate matter SRM 1648a, intact PM and PM deprived of organic compounds (PM∆C). Peritoneal murine macrophages were exposed to different concentrations of PM for 24 h and then challenged with LPS. Production of inflammatory mediators by macrophages was measured to test immunostimulatory/priming capacity of PM.

**Results:**

Particulate matter used at non-cytotoxic concentrations induced a dose-dependent production of proinflammatory cytokines (TNF-α, IL-6, IL-12p40). By contrast, PM∆C were not able to stimulate macrophages. However, macrophages primed with both forms of PM show proinflammatory response upon LPS challenge.

**Conclusions:**

Our data indicate that exposure of macrophages to low concentrations of PM may prime the cells to hyperinflammatory response upon contact with LPS. Further studies are necessary to explain whether the exposure of patients suffering from chronic inflammatory diseases to particulate matter is responsible for the exacerbation of clinical symptoms during bacterial infections.

## Introduction

The last decade has significantly changed the perception of etiopathogenesis of inflammatory and autoimmune disorders (AIDs) such as asthma, chronic obstructive pulmonary disease, rheumatoid arthritis and atherosclerosis [1, 2]. There is substantial evidence proving that chronic inflammation plays a major role in the development and progression of these disorders. Inflammatory response driven by the breakdown of immune tolerance and malfunctioning of the immune system were deemed to constitute a common background for the above-mentioned diseases [3].

Development of these chronic civilization diseases is triggered by a set of factors, both genetic and environmental [4]. Among environmental factors, such as exposure to tobacco smoke, infectious agents, radiation and ultraviolet light exposure, air pollution seems to be specifically involved in the pathogenesis of AIDs [5]. Air pollutants include gases (carbon monoxide, nitrates, sulfur dioxide and ozone), aerosols, as well as particulate matter (PM) that may interact at multiphase interfaces. Primarily, the increased concentration of pollutants in the air has been strongly associated with lung inflammatory diseases [6].

While the coincidence of air pollution and AIDS occurrence has been reported several times, the mechanism behind this relationship is still not clear [7]. Local impact of the inhaled particles seems obvious enough, but how can PM affect other tissues and initiate or aggravate the autoimmune process? Some hypotheses hold the chronic inflammatory process responsible for the pathogenesis of these disorders [8]. Induction of oxidative and nitrosative stress by the inhaled inorganic particles (such as metal oxides) might fuel the chronic inflammation in the lung, cause tissue injury and lead to the generation of oxidatively modified autoantigens. Importantly, it has been documented that alveolar macrophages, the first line of defense of the respiratory tract, engulf PM and secrete a wide array of inflammatory mediators [9].

In general, macrophages, major phagocytes of the immune system, are key cells involved in the regulation of chronic inflammation accompanying numerous infectious and autoimmune diseases [10]. Macrophages, depending on the properties of the stimulant used, may be polarized into distinct functional phenotypes: M1-type proinflammatory/microbicidal cells, M2-type anti-inflammatory/suppressor cells or may acquire a mixed state of activation [11, 12]. Recently, conflicting data have been reported concerning the effects of airborne particulate matter on macrophage activation and polarization [13–15]. All these data explain why we have chosen macrophages as the experimental model cells to be used in vitro to test the influence of PM on innate immunity and inflammatory response [16, 17].

The main aim of this study was to evaluate whether low, nontoxic concentrations of urban PM are able to prime macrophages to altered inflammatory response upon LPS challenge. To fulfill this task, we endeavored to elucidate whether standard urban SRM 1648a samples (PM) and PM∆C, the PM-derived samples significantly devoid of organic content, might affect the viability and secretory functions of the peritoneal murine macrophages. Both direct and priming effects of PM on macrophages were studied. To evaluate the impact of low, non-stimulatory concentrations of PM on the inflammatory response of macrophages, PM-primed cells were challenged with LPS, a potent M1-type, proinflammatory stimulus and a major pathogenic factor of Gram-negative bacterial infections [18].

## Materials and methods

### PM samples

Urban particulate matter samples SRM 1648a (encoded as PM) were purchased from National Institute of Standards and Technology in the United States of America and used as the reference material. The samples were composed of particulate matter collected over a period of 1 year (1976–1977) in the St. Louis (MO) area into a specially designed baghouse. The reference material consists of highest level of iron (Fe) and zinc (Zn) among transition metals [19]. SRM 1648a is a conglomeration of fine and ultrafine particles with the mean particle diameter 5.85 µm. PM contains inorganic elements such as: chlorine (Cl), potassium (K), calcium (Ca), titanium (Ti), vanadium (V) chromium (Cr), manganese (Mn), Fe, nickel (Ni), copper (Cu), Zn, bromine (Br), rubidium (Rb), strontium (Sr) and lead (Pb) (Certificate of analysis Standard Reference Material 1648a Urban Particulate Matter). Moreover, the SRM 1648a contains ca. 13% of carbon, including 10.5% of organic carbon [19]. Plasma Zepto system (Diener electronic GmbH + Co. KG) has been used in our studies to eliminate organic compounds present in the reference material. Samples were treated with low-temperature plasma at highest power for 120 min. Content of carbon was determined by the elementary analysis and total organic carbon analyzer (Schimadzu, TOC-V series). The decreased carbon content from 14% in original samples (PM) to 2% in the plasma-treated PM samples (encoded as PM∆C) has been observed (Mikrut, manuscript in preparation).

Before use, PM and PM∆C particles were weighted on a high precision microbalance and a stock suspension of 2 mg/ml in DPBS (Dulbecco’s phosphate-buffered saline) was prepared. The samples were sonicated for 20 min before use in each experiment.

### Mice

Inbred C57BL/6 male mice (8–12 weeks of age, 18–22 g) were maintained in the Animal Breeding Unit No 2, Faculty of Medicine, Jagiellonian University Medical College, Kraków. All mice were housed in the laboratory room with water and standard diet ad libitum. The authors were granted permission (KRA1_16_2016) by the Local Ethics Committee to use mice in this study.

### Cells: preparation of macrophages

Peritoneal macrophages were induced by an intraperitoneal injection (i.p.) of 3% thioglycollate (1.5 ml per mouse) (Sigma-Aldrich). Macrophages were collected 96 h later by washing out the peritoneal cavity with DPBS. The cells were centrifuged (1500×*g*, 10 min), and red blood cells were lysed by osmotic shock using lysing buffer (155 mM NH_4_Cl, 10 mM NaHCO_3_, 0.1 mM EDTA). At least three mice were used as donors of peritoneal macrophages for each experiment.

### Cell culture and the experimental model

Macrophages were seeded into 24-well flat-bottom cell culture plates (BD) at a density of 5 × 10^5^/well in IMDM (Iscove’s Modified Dulbecco’s Medium) with 25 mM HEPES, supplemented with 5% fetal calf serum (FCS), 2 mM l-glutamine (Cytogen) and 0.04 mg/ml gentamycin (KRKA). After 2 h (CO_2_ incubator, 37 °C), the culture medium containing non-adherent cells and extracellular products of the seeded cells was removed and replaced by the fresh IMDM. Then, the remaining adherent cells (peritoneal macrophages) were used to study the immunostimulatory properties of PM samples in two experimental models. Experiments were repeated at least three times.

A. *Direct immunostimulatory effect of PM* Peritoneal macrophages were incubated with original (PM) or plasma-treated (PM∆C) samples at concentrations ranging from 1 to 400 µg/ml for 24 h. After that time, supernatants were collected and frozen at 80 °C until further use.
B. *Priming effect of PM* Peritoneal macrophages were pre-treated with PM or PM∆C (at concentrations ranging from 1 to 400 µg/ml) for 24 h. After that time, the medium was collected and replaced with the fresh one containing LPS (100 ng/ml). 24 h later (24–48 h of the experiment), supernatants were collected and frozen at 80 °C until further analyses. Cells were studied in a Western blot and flow cytometry analysis.

### Evaluation of cell morphology

Thioglycollate-induced peritoneal macrophages were cultured as described above. After 24 h culture, with or without PM/PM∆C, cells were examined under light microscopy using Axiovert 40CLF inverted microscope (Carl Zeiss). Cells cultured in UpCell 24 Multidish plates (Nunc) were transferred from CO_2_ incubator (37 °C) and kept for 30 min at a room temperature, to allow the detachment of the adherent cells. Then, cells were washed in PBS containing 2% FCS and 0.02% sodium azide and analyzed on Becton Dickinson FACS Calibur with CellQuest Pro Software (BD Biosciences). Cell morphology was measured on FSC/SSC plots after excluding cell debris (gate R1) and then gating on FSC high macrophages (gate R3) and low FSC events (damaged, dying cells) (gate R2).

### Determination of cytokines concentration

Cytokine levels in cell culture supernatants were measured by sandwich ELISA. Microtiter plates (Costar EIA/RIA plates, Corning Inc.) were coated with a cytokine-specific antibody. Expression levels of IL-6, IL-10, and IL-12p40 were measured according to the manufacturer’s instructions (OptEIA Sets, BD Biosciences). TNF-α level was measured according to the manufacturer’s instructions (ELISA Ready-Set-Go, eBioscience). In all cases, 10% FCS in PBS was used as a blocking solution. TMB substrate solution (BioLegend) was used to develop a colorimetric reaction, which was stopped with 2 M sulfuric acid. Optical density was measured at 450 (570) nm using a microtiter plate reader (PowerWaveX, Bio-Tek Instruments).

### Nitrite (NO_2_^−^) determination

The level of nitrites (an oxidative end product of NO) was determined by a microplate Griess assay [20]. Briefly, 100 µl of cell supernatants was incubated with an equal volume of Griess reagent [1% sulphanilamide in 2M HCl (Sigma-Aldrich) and 0.1% *N*-1-naphthylenediamine dihydrochloride in deionized water (POCH)] at room temperature (RT) for 10 min. The absorbance at 550 nm was measured by a microplate reader. Nitrite concentration was calculated from a sodium nitrite standard curve.

### PGE_2_ determination

PGE_2_ concentration in supernatants was determined by Prostaglandin E_2_ Monoclonal EIA kit (Cayman Chemical) according to the manufacturer’s instruction.

### Western blot analysis

Expression levels of COX-2, iNOS, and HO-1 proteins in cell cytosol were determined by Western blot assay in macrophages after PM/PMΔC (24 h) and LPS (24 h) treatment. Upon supernatant collection, cells were lysed in lysis buffer (1% Triton X-100, 0.1% SDS in PBS) containing protease inhibitor cocktail (Sigma-Aldrich). Protein concentrations in lysates were determined using a bicinchoninic acid protein assay kit (Sigma-Aldrich). Samples containing equal amounts of total protein were mixed with gel loading buffer (0.125M Tris, 4% SDS, 20% glycerol, 0.2M dithiothreitol, 0.02% bromophenol blue) at a 2:1 ratio (v/v) and boiled for 4 min. Samples of 20µ g or 10µ g of total protein per lane were separated on 10% SDS–polyacrylamide gels (Biosciences) using the Laemmli buffer system. Proteins were transferred to nitrocellulose membranes (Bio-Rad). Nonspecific binding sites were blocked overnight at 4 °C with 4% non-fat dried milk. Membranes were incubated for 2 h, at a RT with polyclonal antibodies to COX-2 (Cayman), monoclonal antibody to HO-1 1:1000, (Enzo), or iNOS 1:1000, (Enzo). Bands were detected with alkaline phosphatase-conjugated secondary goat antibody to the rabbit IgG whole molecule (1 h at RT, 1:3000), (Sigma-Aldrich) and developed with BCIP/NBT alkaline phosphatase substrate (Sigma-Aldrich). Membranes were re-probed with monoclonal anti β-actin antibody (clone AC-15, 1 h at RT, 1:3000, Sigma-Aldrich). Prestained SDS–PAGE standards (low and high range) (Bio-Rad) were used for molecular weight determinations. Protein bands were scanned and analyzed with the Scion Image freeware (Scion Corp.). Data were normalized to the constitutive expression level of β-actin protein.

### Statistical analysis

Statistical significance of differences between groups was analyzed using one-way ANOVA, followed, if significant, by a Dunnett’s test for post hoc comparison. Results are expressed as mean ± SEM values. A *P* value < 0.05 was considered statistically significant. Analysis was performed using Graphpad Prism v. 5.01 (GraphPad Software, Inc.).

## Results

### The effect of PM and PMΔC on cell morphology and viability

**Fig. 1:**
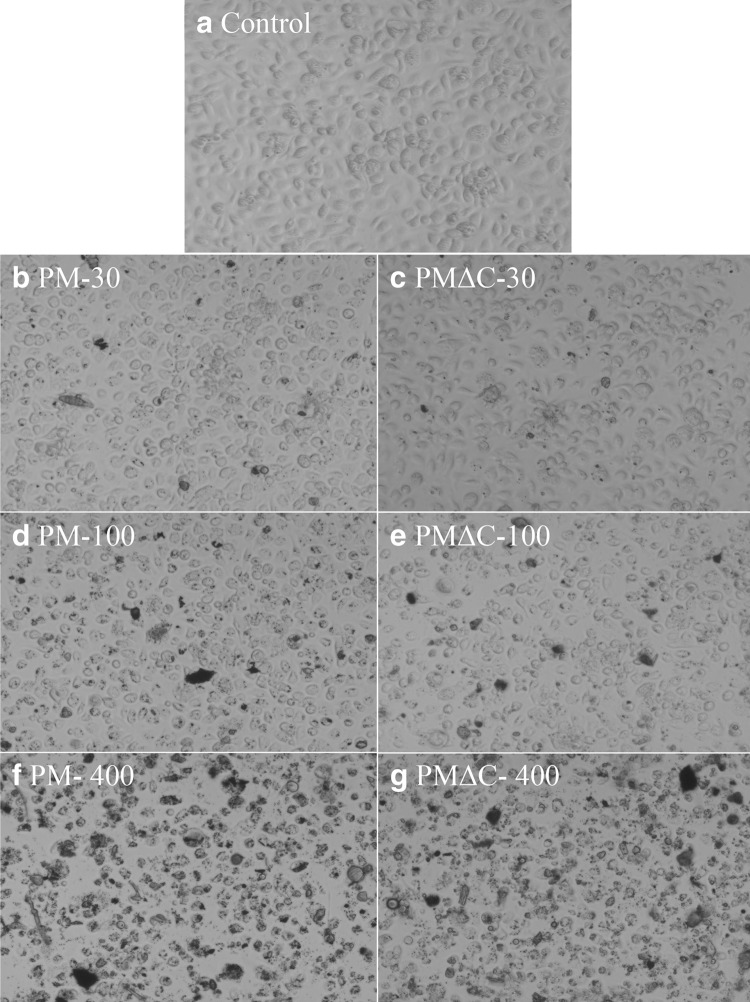
Microscopic images (inverted microscope, ×20 magnification) of macrophages cultured in vitro with PM or PM∆C. Mouse peritoneal macrophages were cultured in vitro for 24 h in the presence of PM at concentrations of 30 µg/ml (**b**), 100 µg/ml (**d**), 400 µg/ml (**f**) or PM∆C at concentrations of 30 µg/ml (**c**), 100 µg/ml (**e**), 400 µg/ml (**g**) or in medium only (**a**—control, untreated cells). The images are representative pictures of cell cultured in at least three independent experiments run in three experimental replicates

**Fig. 2:**
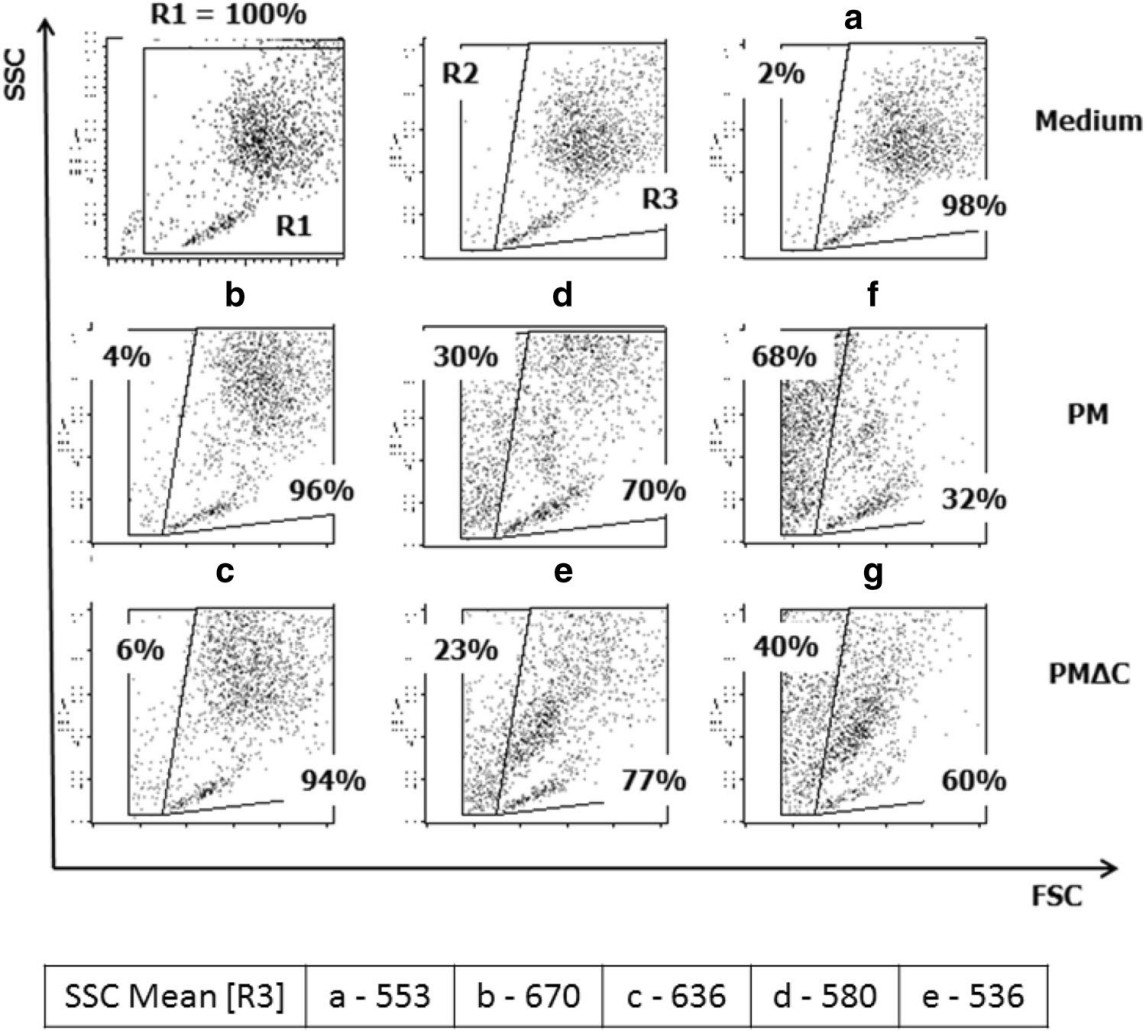
Morphological changes in peritoneal exudate cells cultured in vitro in the presence of PM or PM∆C shown as dot plot images of the cell size (FSC) vs internal complexity (SSC). Cells were cultured in vitro for 24 h in the presence of PM (middle panel) at concentrations of 30 µg/ml (**b**), 100 µg/ml (**d**), 400 µg/ml (**f**) or PM∆C (lower panel) at concentrations of 30 µg/ml (**c**), 100 µg/ml (**e**), 400 µg/ml (**g**). Negative control cells were cultured in medium only (**a**). Cells within gate R1 were analyzed (R1 = 100%). Percentage of small (low FCS) cells (gate R2) and big (high FCS, normal for macrophages) (gate R3) are shown. The data was obtained from three independent experiments run in three technical replicates

Morphological features of cells exposed in vitro to PM or PMΔC were examined by light microscopy (Fig. 1) and flow cytometry (Fig. 2). To evaluate direct influence of PM and PMΔC on peritoneal macrophages, cells were cultured in the presence of various concentrations (1–400 µg/ml) of the PM/PMΔC for 24 h. Corpuscular nature of the tested samples causes that only a fraction of the cells in culture is in direct contact with PM/PMΔC used at low concentrations < 100 µg/ml (< 60 µg/cm^2^). However, some differences between microscope images of PM and PMΔC can be observed. As shown in Fig. 1b–e, PM at concentrations of 30 and 100 µg/ml was accumulated by a larger numbers of macrophages than PMΔC used at the same concentrations. It indicates that PM samples are engulfed more readily than PMΔC ones.

This phenomenon was confirmed using flow cytometry (Fig. 2). Analysis of cell size (FSC) vs cell granularity (SSC) revealed that in the presence of PM or PMΔC at a concentration of 30 µg/ml the SSC parameter has increased (Fig. 2b, c) compared to control untreated cells (Fig. 2a). SSC Mean values have risen from 553 (Fig. 2a, control) to 670 (PM 30 µg/ml, Fig. 2b) and 636 (PMΔC 30 µg/ml, Fig. 2c). The difference between PM (SSC mean value = 580) and PMΔC (SSC mean value = 536) is more pronounced at a concentration of 100 µg/ml (Fig. 2d, e). These data suggest that engulfment of PMΔCs is less effective compared to PM. High concentrations of tested samples (above 100 µg/ml) had detrimental impact on cells in the culture. PM at 400 µg/ml caused cell injury, as a large amount of debris is visible (Fig. 1f). Damage of the cells is depicted in flow cytometry dot plots (Fig. 2f, g). The percentage of cells in gate R3 decreased from 98% (control, Fig. 2a) to 32% when cells were treated with PM at concentration of 400 µg/ml (Fig. 2f). The effect of PMΔC (Fig. 2g) was less definite. These data imply that both PM and PMΔC are cytotoxic for macrophages when used at concentrations above 100 µg/ml. It is in agreement with other reports [21].

### Direct impact of PM and PMΔC on macrophage secretory functions

**Fig. 3:**
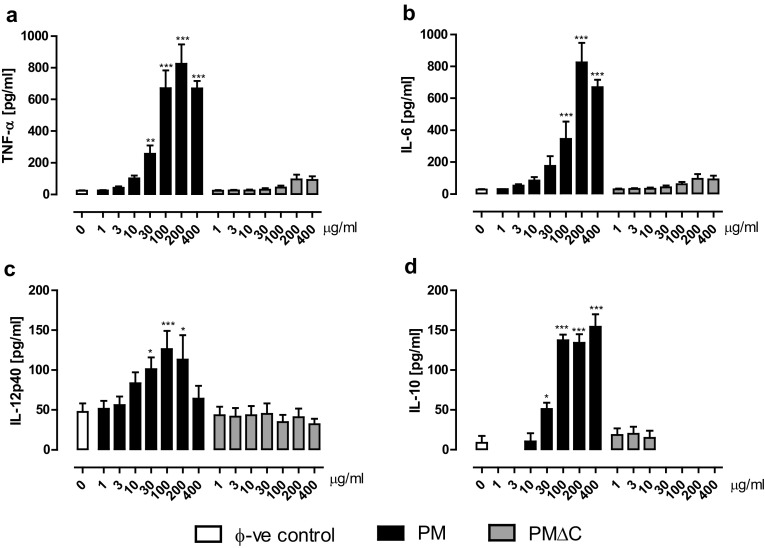
Production of inflammatory mediators from mouse peritoneal macrophages after in vitro stimulation with PM and PM∆C. Cells were stimulated with different concentrations of either PM or PM∆C. Supernatants were collected after 24 h and the concentrations of TNF-α (**a**), IL-6 (**b**) IL-12p 40 (**c**) and IL-10 (**d**), were determined as described in methods. Data are mean ± SEM value of three independent experiments run in three technical replicates. *P < 0.05, **P < 0.005, ***P < 0.0001, PM/ PM∆C-treated vs untreated macrophages

To evaluate the immunostimulatory effect of particulate matter on functioning of the inflammatory cells, supernatants were collected after 24 h incubation of peritoneal macrophages with increasing concentrations of PM or PMΔC. For the original PM samples, a dose-dependent enhancement of synthesis of all the tested cytokines (TNF-α, IL-6, IL-12p40 and IL-10) was observed (Fig. 3). The most potent effect was found at concentrations 100–200 µg/ml. Importantly, the similar enhancement of synthesis of major proinflammatory (TNF-α) and anti-inflammatory (IL-10) cytokines was achieved. On the contrary, macrophages stimulated with PMΔC, used at the same concentrations, did not secrete any of the tested cytokines. These data suggest that PM with lowered content of organic components lost their ability to directly induce inflammatory response of macrophages.

### Priming effect of PM and PMΔC on macrophages restimulated with LPS

To determine the impact of PM and PMΔC on macrophage state of activation, the cells were pre-incubated (primed) with various concentrations of either PM or PMΔC and then activated with LPS which induces M1-like pattern of proinflammatory mediators [18].

**Fig. 4:**
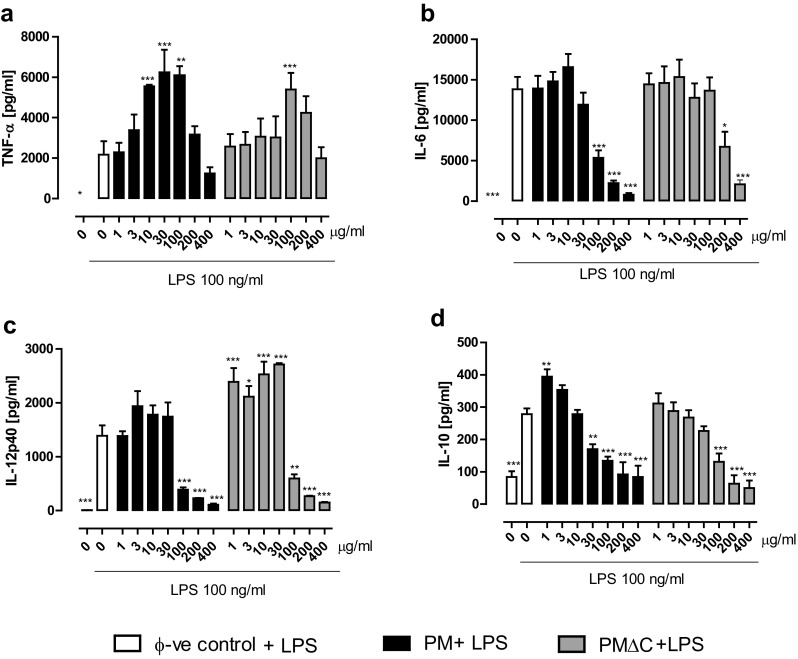
Priming effect of PM and PMΔC on cytokine secretion: TNF-α (**a**), IL-6 (**b**), IL-12p40 (**c**), IL-10 (**d**) by peritoneal macrophages. Cells (5 × 10^5^ per well) were pre-incubated with PM samples for 24 h (at indicated concentrations from 1 to 400 µg/ml) and then stimulated with LPS (100 ng/ml) for another 24 h. After that time, supernatants were collected and the concentrations of cytokines were estimated by ELISA. Data are mean ± SEM value of three independent experiments run in three technical replicates. Control group—stationary macrophages stimulated with LPS only after 24 h culture in a standard medium. *P < 0.05, **P < 0.01, ***P < 0.001 control macrophages (stimulated with LPS) vs macrophages primed with indicated concentrations of PM and then stimulated with LPS 100 ng/ml

**Fig. 5:**
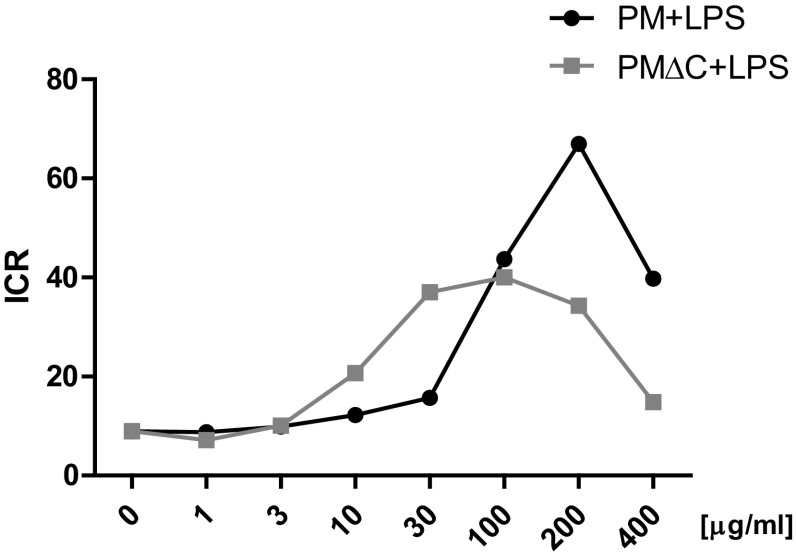
Inflammatory cytokine ratio of macrophages upon PM/PMΔC treatment with subsequent LPS stimulation. Macrophages were pre-incubated with PM samples for 24 h (at concentrations ranging from 1 to 400 µg/ml) and then stimulated with LPS (100 ng/ml) for another 24 h. Inflammatory ratio is defined as TNF-α/IL-10 concentrations ratio in supernatants collected after the experiment. Data was derived from three independent experiments run in three technical replicates

In this experimental setting, we observed significantly reduced production of cytokines (IL-6, IL-12p40, IL-10) by macrophages primed with high concentrations of PM and PMΔC (> 100 µg/ml). The decrease of TNF-α secretion was not statistically significant (Fig. 4). By contrast, PM used at low concentrations (10–100 µg/ml) was able to enhance the secretion of cytokines by macrophages restimulated with LPS (Fig. 4). Most importantly, TNF-α secretion was significantly increased upon priming with 10–100 µg/ml for PM and 100 µg/ml for PMΔC. Concurrently, both PM and PMΔC gradually decreased the production of IL-10. In addition, to evaluate the proinflammatory state of activation of primed macrophages, we have created the inflammatory cytokine ratio (ICR, Fig. 5). ICR, calculated as a ratio of TNF-α to IL-10 concentration, increased from ca. 9 in control group (macrophages stimulated with LPS) to above 40 in macrophages primed with both PM and PMΔC and restimulated with LPS.

**Fig. 6:**
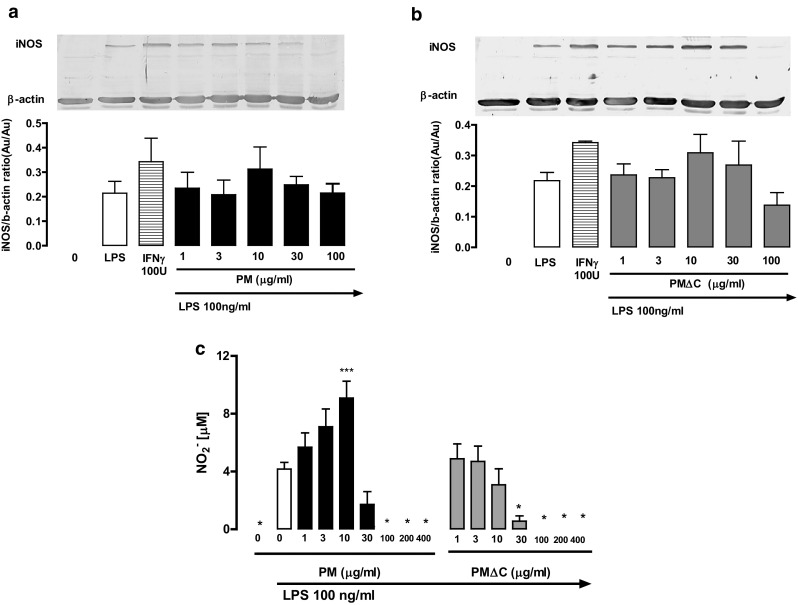
Influence of PM and PMΔC on the expression of iNOS and the production of nitrites by peritoneal macrophages cultured in vitro. The expression of iNOS by cells (5 × 10^5^ per well) cultured in vitro for 24 h in the presence of PM (**a**) or PM∆C (**b**) at indicated concentrations (1–100 µg/ml) is shown and then stimulated with LPS (0.1 µg/ml) for another 24 h. IFN-γ was used a positive control for iNOS production in macrophages. Representative blot picture is shown as well as the quantitative Western blot data derived from three independent experiments, normalized to constitutively expressed β-actin protein ± SEM (no statistically significant changes were observed). The concentration of nitrites (NO_2_^−^) into the culture supernatant (**c**) upon LPS stimulation (for 24 h) of cells pre-treated with PM or PM∆C (for 24 h) was measured by Griess method. *P < 0.05, ***P < 0.001 control macrophages (stimulated with LPS) vs macrophages primed with indicated concentrations of PM and then stimulated with LPS 0.1 µg/ml. Data are mean values ± SEM from three independent experiments run in three technical replicates

**Fig. 7:**
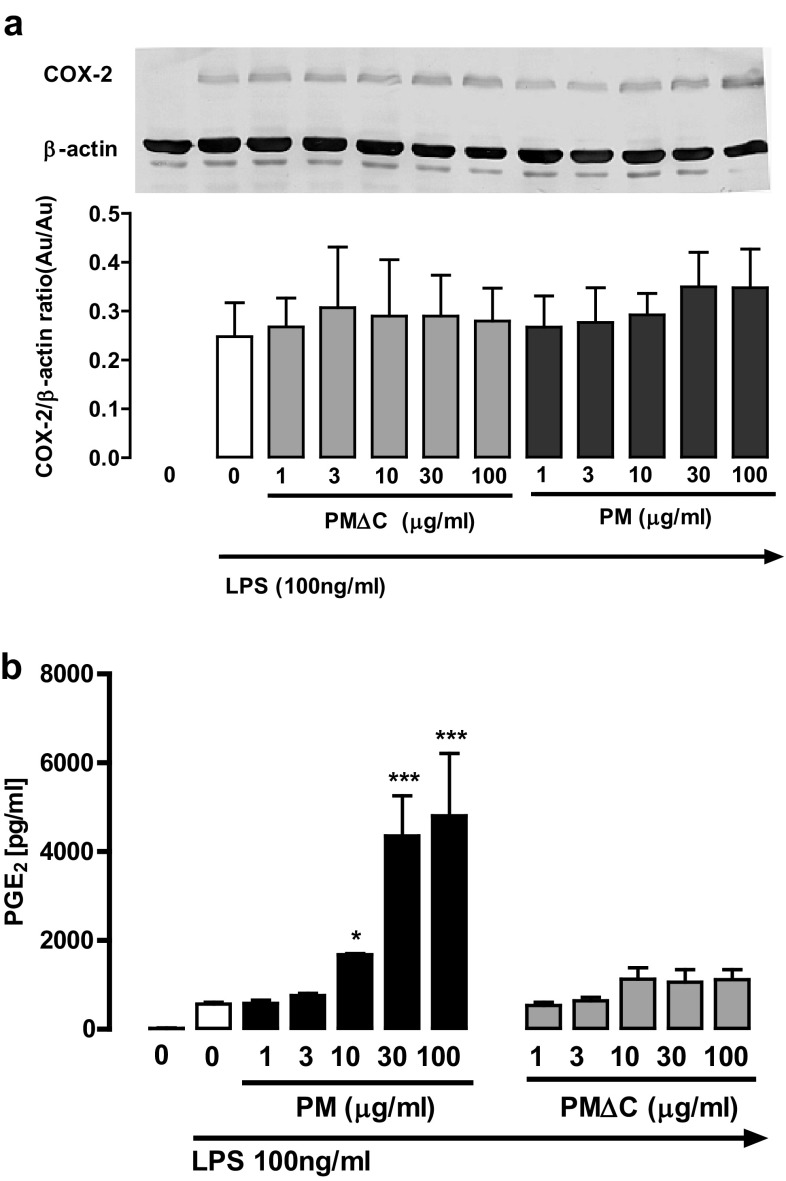
Influence of PM and PMΔC on the COX-2 expression and production of PGE_2_ by peritoneal macrophages cultured in vitro. The expression of COX-2 (**a**) and release of PGE_2_ into the culture supernatant (**b**) by cells (5 × 10^5^ per well) cultured in vitro for 24 h in the presence of PM or PM∆C at indicated concentrations (1–100 µg/ml) and then stimulated with LPS (0.1 µg/ml) for another 24 h. Representative blot picture is shown as well as the quantitative Western blot data derived from three independent experiments, normalized to constitutively expressed β-actin protein ± SEM (no statistically significant changes were observed). The release of PGE2 into the culture supernatant (**b**) upon LPS stimulation (for 24 h) of cells pre-treated with PM or PM∆C (for 24 h) was measured using ELISA. *P < 0.05, ***P < 0.001 control macrophages (stimulated with LPS) vs macrophages primed with indicated concentrations of PM and then stimulated with LPS 100 ng/ml. Data are mean values ± SEM from three independent experiments run in three technical replicates

We also evaluated the generation of PGE_2_, the major eicosanoid produced by macrophages at a site of inflammation, and the release of NO, the key microbicidal agent of activated macrophages [22–24]. The concentrations of PGE_2_ and NO were elevated in macrophages primed with PM (10–100 µg/ml) but not with PMΔC (Figs. 6c, 7b). Interestingly, NO secretion reached its peak at 10 µg/ml of PM with very low levels in case of higher PM concentrations.

Importantly, the production of PGE_2_ and NO did not correlate with the level of enzymes responsible for their production. The expression of COX-2 and iNOS in macrophages primed with PM/PMΔC and challenged with LPS was similar with that detected in macrophages stimulated only with LPS, while PM/PMΔC trained macrophages showed the altered production of PGE_2_/NO (Figs. 6a, b, 7a).

## Discussion

Exposure to the air particulate matter is epidemiologically associated with various chronic inflammatory and autoimmune diseases. Notably, increased incidence and exacerbation of cardiopulmonary disorders has been found to associated with elevated levels of urban particulate matter [6, 25, 26]. Respirable particles reach the lower respiratory tract where they are phagocytosed by alveolar macrophages leading to a local inflammation [14]. The main current hypothesis is that the particles produce pulmonary inflammation with systemic release of macrophage-driven cytokines, which may influence remote cardiovascular and central nervous system endpoints [27–29]. Nevertheless, there are conflicting and inconsistent data in the literature proving the opposing effects of various PM samples on the inflammatory response of macrophages. Numerous reports of studies on macrophages exposed to a wide spectrum of particulate matter coming from ambient air pollution provides examples of enhanced proinflammatory activity of macrophages, increased apoptosis and necrosis as well as autophagy levels [21, 30, 31]. Moreover, various mechanisms have been proposed to explain the exacerbating effect of particulate matter on the inflammatory response. First, the generation of reactive oxygen and nitrogen species might lead to development/exacerbation of inflammatory response via oxidative/nitrosative stress induction. This effect might be enhanced by the presence of transition metals (mostly as oxides) triggering the Fenton’s reaction and generation of ROS [32]. Second, it seems possible that proinflammatory properties of particulate matter might be attributed to the LPS contamination of the samples, as described in some reports [33]. On the other hand, there are also data on anti-inflammatory action of PM leading to suppression of the proinflammatory macrophage phenotype [14, 34]. In addition, the particulate matter has been shown to induce polarization of macrophages towards opposing M1 and M2 phenotypes [35, 36].

What would be the underlying cause of such conflicting, discrepant effects described in the literature? It is plausible that the composition of the PM samples used in different studies may vary significantly leading to various effects in the experimental models employed [14, 37]. Indeed, air pollution particulate matter is a conglomeration of many components (metals, sulfate, nitrates, hydrocarbons and endotoxin) showing distinct biological activities. Therefore, either application of advanced chemical analysis of particulate matter samples seems necessary prior to cellular/animal studies to allow subsequent comparisons of the results from different research groups or standard reference material of particulate matter should be used. To address the above suggestions, in the present study we used the standard reference urban air PM (SRM 1648a) to test its influence on inflammatory response. To examine a direct and a priming effect of PM on secretory properties of murine peritoneal macrophages, two experimental models have been used, as described in the methods. Importantly, two forms of the SRM 1648a particles were studied to estimate the impact of organic compounds on their activities. Namely, intact original PM (the conglomerate of both organic and inorganic compounds) and PM deprived of carbon compounds (PMΔC, containing mainly inorganic, e.g., transition-series metals compounds) were examined at concentrations of 1–400 µg/ml. Such wide-range concentrations of PM provides low (non-active), moderate (stimulatory), as well as high (cytotoxic) amounts of the tested compounds.

In concordance with other studies [21, 38], our data have shown the toxic effect of SRM 1648a (PM and PMΔC) used at concentrations above 100 µg/ml (equivalent of ~ 60 µg/cm^2^). Microscopic evaluation revealed the reduced cell count and altered macrophage morphology as compared with the control (see the “Results” section). Moreover, cytometric analysis confirmed that both cell size and morphology changed in PM-treated cells. All the effects mentioned above were less pronounced in the case of PMΔC which points to the importance of the organic fraction of the air pollution components for the cytotoxic effect. This observation is inconsistent with the hypothesis that mostly inorganic compounds such as transition-metal oxides (ferric, manganese oxides, etc.) are responsible for the particulate matter cytotoxicity [39]. However, the observed phenomenon might be limited to the SRM particles as we could not observe ROS generation in our experimental set-up (data not shown). On the other hand, we have observed massive release of cytokines.

Indeed, significant increase of all tested cytokines levels—both proinflammatory TNF-α, IL-6, IL-12p40 and anti-inflammatory IL-10—was observed upon incubation of macrophages with high concentrations (100–400 µg/ml) of the original PM samples. It indicates that macrophages exposed to cytotoxic concentrations of PM are able to produce cytokines before they die. It may be explained by a distinct rate of cytokine production and PM-caused cell death (e.g., very fast production of TNF-α vs cell death via necrosis or apoptosis) [40]. In addition, dying cells might release damage-associated molecular patterns (DAMPs) that could then trigger neighboring, still alive macrophages, to produce the inflammatory mediators [41]. However, the exposure of macrophages to PMΔC negates the latter mechanism, as even cytotoxic concentrations of PMΔC did not evoke the production of any tested cytokine. Therefore, further studies are necessary to address the issue why PM deprived of carbon compounds loses its immunostimulatory properties.

In the next stage of our studies, we examined the priming properties of the SRM 1648a particles. It seems plausible that the effects of particulate matter might not rely on the direct impact of PM components on the cells governing the inflammation. Therefore, our studies were aimed at the evaluation of how the macrophage response to proinflammatory stimulus such as LPS might be affected if the cells were pre-exposed to particulate matter. Interestingly, we observed that the priming with original PM (at 10, 30 and 100 µg/ml) induced significantly increased secretion of TNF-α upon subsequent LPS stimulation. Moreover, the analysis of the ratio of TNF-α to IL-10 levels (inflammatory cytokine ratio) revealed that higher concentrations of both PM and PMΔC correlated with the increase of proportion of the pro- to anti-inflammatory cytokine concentrations. The studied inflammatory cytokine ratio increased with the rising concentrations of both PM and PMΔC up to 100 µg/ml. On the other hand, ICR decrease at the higher concentrations is most probably the effect of the reduced cell viability upon incubation with such high doses of particulate matter. Despite similar ICR of macrophages primed with both PM and PMΔC, some differences in the pattern of secreted mediators were observed. Namely, macrophages primed with PM exhibit enhanced secretion of TNF-α, while IL-12p40 levels were found significantly increased when cells were pre-treated with PMΔC.

Interestingly, the enhancement of PGE_2_ and NO production did not correlate with the levels of enzymes responsible for their production. The expression of COX-2 and iNOS in macrophages primed with PM/PMΔC and challenged with LPS was similar with that detected in macrophages stimulated only with LPS. This might point to different mechanisms of regulation of PGE_2_/NO production in non-trained and PM-trained (primed) macrophages. The increase in PGE2 production without significant changes in the COX-2 expression suggests that macrophages pre-treated with PM and then stimulated with LPS selectively produce PGE_2_ with a concomitant limited production of other COX-2 products (TXA2, PGD2, PGI2, PGF2α). The differential regulation of PGE2, PGD2 and TXA2 (thromboxane A2) production in macrophages has been demonstrated [42, 43]. However, to confirm such regulation in our experimental system the concentration of mPGES-1, the specific PGE2 synthase, should be determined in further studies [44].

On the other hand, the altered production of NO without changes of iNOS expression may be explained by the regulatory properties of PGE2. It has been shown that low concentrations of PGE2 stimulate while high concentrations inhibit the production of nitric oxide [45]. Moreover, in this experimental model, we inferred about NO production from the concentration of nitrites (NO_2_^−^). Therefore, without more advanced analytic studies, it is impossible to exclude the presence of other NO/NO_2_^−^ breakdown intermediates resulting from formation of end products other than the nitrites [46].

All these results suggest that exposure to PM/PMΔC could prime the macrophages for further hyperinflammatory response to other stimuli such as bacterial TLR ligands (e.g., LPS, the ligand for TLR4). Such phenomenon characterized by macrophage priming with one agent and an increased cytokine production upon restimulation with the other, is termed “trained immunity” [47, 48]. Importantly, the presence of organic compounds seems to be important in macrophage training with airborne PM.

Taken together, our present study points to a conclusion that the direct immunostimulatory effect of PM containing both inorganic and organic fraction, is mostly associated with the presence of cytotoxic concentrations of carbon compounds. In our experimental set-up, using the reference SRM 1648a particles, such effect was observed above the PM critical concentration of 100 µg/ml. Nevertheless, these data do not rule out a role of transition metals present in other airborne PM in the induction of oxidative stress.

On the other hand, low concentrations of PM neither affected macrophage viability nor their function, as measured by the production of inflammatory mediators. However, PM could act as a priming agent for macrophages subsequently exposed to proinflammatory stimulus. Such trained macrophages became more sensitive to LPS stimulation which results in an amplified state of activation, characteristic for the proinflammatory M-1 type macrophages (inferred from TNF-α/IL-10 ratio analysis). This effect was not restricted to the activity of organic compounds as ICR index was similar for PM and PMΔC. It indicates that exposure of the immune cells to the particulate matter may be responsible for maintaining/exacerbation of chronic inflammation (high TNF-α levels) and for M1-Th1 type responses (low IL-10 levels). Importantly, IL-10 has been identified as a major player in the regulation of Th1 immune responses. Thus, too much IL-10 may suppress protective T cell responses, while too little IL-10 may contribute to the development/aggravation of autoimmune disorders through an excessive activation of T cells [49].

Finally, our data suggest that exposure to even low concentrations of PM containing both inorganic and organic compounds may reprogram (train) macrophages towards the cells responding in an uncontrolled hyperinflammatory reaction upon bacterial infections. Therefore, it may explain association of the increased level of ambient air PM with exacerbation of chronic inflammatory and autoimmune disorders [8]. Moreover, a demonstration of lasting activity of such trained macrophages in a long-time experiments in vivo will support the idea concerning innate immune memory [50]. Nevertheless, further ex vivo studies with human alveolar macrophages exposed to PM are necessary to prove that results from the in vitro experimental animal models are relevant for human medicine.
